# Carcinosarcoma of the Cervix: A Case Report

**DOI:** 10.31729/jnma.6566

**Published:** 2021-08-31

**Authors:** Birendra Bhagat, Bijaya Chandra Aacharya, Sarita Gurung, Ranjan Raj Bhatta, Aasiya Rajbhandari

**Affiliations:** 1 Department of Gynecological Oncology, National Academy of Medical Sciences, Kathmandu, Nepal; 2 Department of Gynecological Oncology, B. P. Koirala Memorial Cancer Hospital, Bharatpur, Nepal; 3 Department of Pathology, B. P. Koirala Memorial Cancer Hospital, Bharatpur, Nepal; 4 Department of Pathology, Paropkar Maternity and Women's Hospital, Kathmandu, Nepal

**Keywords:** *carcinosarcoma*, *cervix*, *sarcoma*, *surgery*, *treatment*

## Abstract

Cervical carcinoma is the most common cause of mortality due to cancer in Nepal. Carcinosarcoma is a very rare subtype of cervical cancer which is characterized by the presence of both epithelial and mesenchymal malignant component. It constitutes less than 1% of cervical carcinoma. Due to the low occurrence of the disease, most of the data on treatment and prognosis are based on case reports and series. Here, we report a case of 69 years, female with cervical cancer (FIGO IIA2). Histopathological and immunohistochemical analysis of cervical biopsy initially showed primary adenosarcoma of the cervix. The tumor was non-responsive to primary treatment with concurrent chemoradiation. Later she was treated with abdominal hysterectomy and bilateral salpingo-oophorectomy. The final histopathology of the resected specimen showed a sarcomatous component along with carcinomatous changes in the endocervical glands favouring the diagnosis of carcinosarcoma of the cervix.

## INTRODUCTION

Cervical carcinoma is the most common carcinoma of females in Nepal and, also the leading cause of mortality occurring from cancer.^1^ Carcinosarcoma mostly arises from the uterine corpus followed by the ovary and the cervix. Carcinosarcoma arising from the cervix is extremely rare and constitutes less than 1% of cervical malignancy.^2^ Carcinosarcomas are mixed tumors characterized by the presence of both epithelial and mesenchymal malignant components. There are only a few reported cases of primary carcinosarcoma of the cervix so, the pathogenesis, treatment and prognosis have not been clearly outlined. Here we report a case of carcinosarcoma of the cervix.

## CASE REPORT

**Figure 1:**
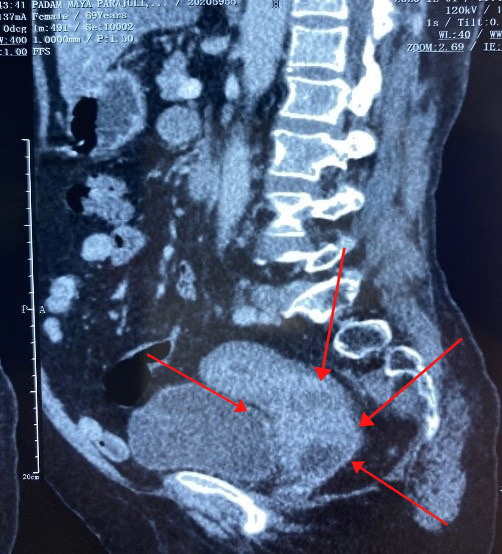
CT scan showing 9x6x5 cm involving the cervix.

**Figure 2:**
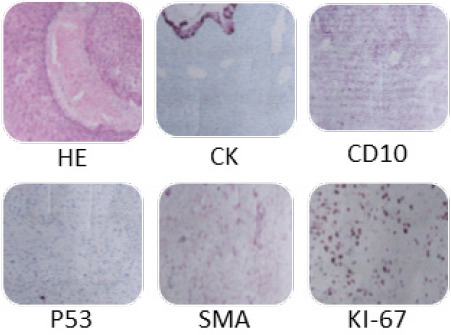
IHC of cervical biopsy tissue.

A 69-year-old female presented to the B.P Koirala Memorial Cancer Hospital with complaints of per-vaginal foul-smelling discharge and intermittent bleeding for 3 months associated with lower abdominal pain. She was multiparous and postmenopausal for 20 years. She was a non-alcoholic and nonsmoker. She never had undergone any cervical screening for cancer detection. Her intermittent bleeding per-vagina brought her to seek medical advice in a nearby health facility where she was suspected to have cervical cancer. On general examination, she was pale with tachycardia. Other vitals were stable with a Body Mass Index (BMI) of 23.4 kg/m2. The cervix was totally replaced by a cauliflower-like growth measuring 8 x 6 cm with involvement of the upper vagina. Bilateral parametria was not involved and rectal mucosa was free. The disease was clinically staged as FIGO stage IIA2. A punch biopsy was taken from the tumor growth. Initially, a CT scan was planned which showed a heterogeneously enhancing space-occupying lesion of size 9 x6 x5 cm involving the cervix and lower uterine segment. The mass abutted the anterior rectal wall posteriorly, and the posterior urinary bladder wall anteriorly. However, no intraluminal extension was seen on either side. There was no lymphadenopathy (Figure 1). Histopathology of the biopsy specimen showed a tumor composed of sheets of spindle cells with moderate pleomorphism with interspersed glands lined by benign endocervical type epithelium. Immunohistochemistry favoured to the diagnosis of primary adenosarcoma of cervix. Benign epithelial lining showed positive reaction with cytokeratin (CK). The sarcomatous component was weakly immunoreactive for SMA (smooth muscle antigen), CD10, P53, P63, while it was negative for desmin, P40, S-100 and ER (Figure 2). Ki67 proliferation index was 20-30%.

**Figure 3:**
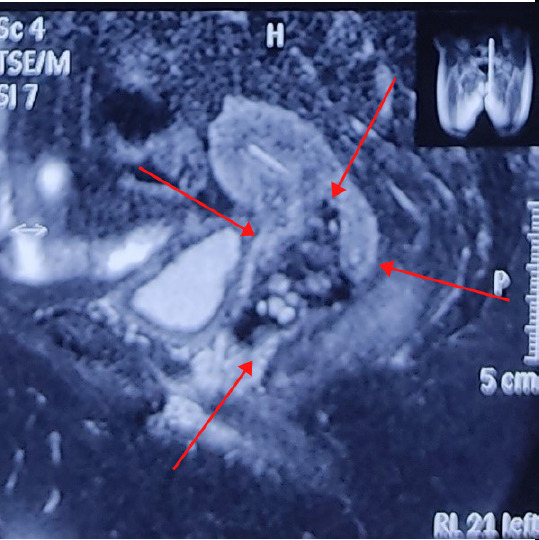
MRI showing cervical mass of size 7.2 x5.6x4.5cm.

**Figure 4:**
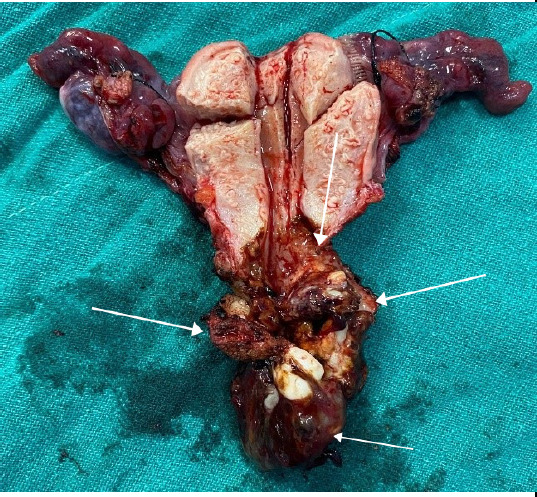
Hysterectomy specimen showing cervical tumor.

Her initial hemoglobin was 7.4 gm%. She was transfused two pints of whole blood and hemoglobin raised to 11.2 gm%. Her blood sugars were also deranged, fasting blood sugar was 168 mg/dl and post-prandial 301 mg/dl. She was diagnosed to have type 2 diabetes mellitus. She was started on insulin therapy and metformin. Her blood sugars were normalized in a week. Her other investigations like chest x-ray, liver function test, serum electrolytes, urine RE/ME, HbsAg, HIV, HCV, VDRL, and ECG were normal. She was planned for concurrent chemoradiation for her bulky cervical tumor after taking an opinion from a medical oncologist and radiotherapist. She received 48 grey/24 fractions of External Beam Radiotherapy (EBRT) concurrent with weekly dose of inj. Carboplatin (AUC 2) for 4 cycles. Inj. Carboplatin was preferred over Inj. Cisplatin as her serum creatinine level was increased. Intracavitary radiotherapy was not possible due to bleeding and the persistence of mass. A booster dose of 14 grey/ 7 fractions of EBRT was again added to the tumor growth which showed no response. A repeat imaging was planned and MRI showed the tumor with no response to the therapy. The size of the tumor was 7.2 x 5.6 x 4.5 cm (Figure 3). She was then planned for surgery. She underwent total abdominal hysterectomy and bilateral salpingo-oophrectomy (Figure 4). There were no palpable pelvic lymph nodes or metastatic deposits in the pelvic peritoneum.

**Figure 5:**
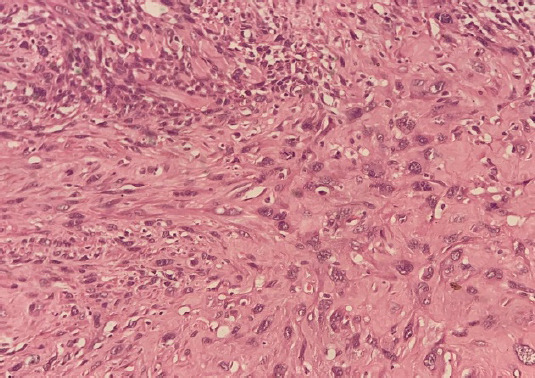
Showing malignant epithelial and mesenchymal component (H&E x 400).

Her final histopathology report of the specimen showed a sarcomatous component along with carcinomatous changes in the endocervical glands (Figure 5). Thus, a final histopathological diagnosis of carcinosarcoma of the cervix was made. Her resected specimen showed tumor-free margins with no parametrium and lymphovascular space invasion. Her post-operative period was uneventful and she was discharged on the 7^th^ day of surgery. Its four months following surgery and she is presently on routine follow-up with no signs and symptoms of recurrence.

## DISCUSSION

Carcinosarcoma of the cervix can originate from either the mesonephric duct or paramesonephric duct. Carcinosarcomas that arise from the uterine cervix are biphasic and contain both carcinomatous(epithelial) and sarcomatous(mesenchymal) malignant components. Carcinosarcoma is more common in the uterine corpus than in the uterine cervix. The uterine origin can be differentiated from the cervical origin by its location, histopathology and immunohistochemistry.^3^ The incidence of cervical carcinosarcoma was 0.48% in a large study done by Bansal, et al.^4^ The most common symptom is vaginal bleeding and discharge.^3^ It usually occurs in post-menopausal women and the median age of diagnosis of carcinosarcoma is 64 years.^4^ The etiopathogenesis is not well understood. Some of the etiological factors are advanced age, previous radiation exposure, chemotherapy and HPV infection.^5^ Differential diagnoses of carcinosarcoma of the cervix are differentiated and undifferentiated carcinomas and sarcomatoid carcinoma. Immunohistochemistry studies help with the identification of both epithelial and mesenchymal components. The biological behavior of cervical carcinosarcomas is presently unclear due to their rarity and the limited or absence of follow-up of many patients. In case of uterine carcinosarcoma, this tumour spreads through the lymphatics, and metastases are usually of the epithelial component. The common sites for tumor metastasis in uterine carcinosarcoma are the pelvic and paraaortic lymph nodes, peritoneum and the lungs.^6^ Recurrence to peritoneum, liver and lungs has also been described in carcinosarcoma of the cervix after radical hysterectomy in a case report.^7^

The treatment modality for carcinosarcoma is not well defined, and limited to case reports only. Laterza, et al.^7^ described the treatment modality and prognosis of 33 patients with cervical carcinosarcoma in the year 2007. Early stages were treated with surgery while advanced stages were treated with radiotherapy. 50% of patients with the early-stage disease were tumor-free at a median follow-up of 16 months. Two out of three patients with stage II were treated by surgery and radiation. One out of three patients with stage II were treated by radiation alone. Two had no evidence of disease and one died of the disease. Comert, et al.^8^ conducted a review of case series and case reports. They included 81 patients with carcinosarcoma and reported that in patients with advanced disease, cytoreductive surgery to remove all macroscopic diseases should be preferred. Ribeiro, et al. reviewed a case report of 11 patients with mesonephric carcinosarcoma of the cervix.^9^ Most of the patients had stage IB at diagnosis, three were stage IIA, one IIIB and one IVB. All patients underwent total hysterectomy with bilateral salpingo-oophorectomy as primary treatment. Primary surgery followed by adjuvant radiotherapy alone or with chemotherapy has been associated with improved survival.

Recently in 2020, Sun, et al. reported a case of cervical carcinosarcoma stage IIIB in which epithelial malignant component responded to radiotherapy but persisting sarcomatous component required hysterectomy and bilateral oophorectomy.^10^ In our case, the patient was primarily treated with chemoradiation as it was a FIGO stage IIA2 bulky disease. Since the tumor did not respond to initial treatment, hysterectomy with bilateral oophorectomy was done. The patient is under follow-up and disease-free till two months following surgery.

In conclusion, primary carcinosarcoma of the cervix is a very rare subtype of cervical malignancy. The need for immunohistochemical analysis for its diagnosis can't be denied. Most of the cases have been treated with primary surgery and we recommend the same as our case didn't respond to initial chemoradiation. Other treatment options are radiotherapy and chemotherapy but further shreds of evidence are required to access its effectiveness and safety.
